# Depression, Comorbid Anxiety Disorders, and Heart Rate Variability in Physically Healthy, Unmedicated Patients: Implications for Cardiovascular Risk

**DOI:** 10.1371/journal.pone.0030777

**Published:** 2012-02-15

**Authors:** Andrew H. Kemp, Daniel S. Quintana, Kim L. Felmingham, Slade Matthews, Herbert F. Jelinek

**Affiliations:** 1 School of Psychology, University of Sydney, Sydney, New South Wales, Australia; 2 School of Psychology, University of New South Wales, Sydney, New South Wales, Australia; 3 School of Medical Sciences, University of Sydney, Sydney, New South Wales, Australia; 4 Centre for Research in Complex Systems, Charles Sturt University, Albury, New South Wales, Australia; 5 Australian School of Advanced Medicine, Macquarie University, Sydney, New South Wales, Australia; Chiba University Center for Forensic Mental Health, Japan

## Abstract

**Context:**

There is evidence that heart rate variability (HRV) is reduced in major depressive disorder (MDD), although there is debate about whether this effect is caused by medication or the disorder per se. MDD is associated with a two to fourfold increase in the risk of cardiac mortality, and HRV is a robust predictor of cardiac mortality; determining a direct link between HRV and not only MDD, but common comorbid anxiety disorders, will point to psychiatric indicators for cardiovascular risk reduction.

**Objective:**

To determine in physically healthy, unmedicated patients whether (1) HRV is reduced in MDD relative to controls, and (2) HRV reductions are driven by MDD alone, comorbid generalized anxiety disorder (GAD, characterized by anxious *anticipation*), or comorbid panic and posttraumatic stress disorders (PD/PTSD, characterized by anxious *arousal*).

**Design, Setting, and Patients:**

A case-control study in 2006 and 2007 on 73 MDD patients, including 24 without anxiety comorbidity, 24 with GAD, and 14 with PD/PTSD. Seventy-three MDD and 94 healthy age- and sex-matched control participants were recruited from the general community. Participants had no history of drug addiction, alcoholism, brain injury, loss of consciousness, stroke, neurological disorder, or serious medical conditions. There were no significant differences between the four groups in age, gender, BMI, or alcohol use.

**Main Outcome Measures:**

HRV was calculated from electrocardiography under a standardized short-term resting state condition.

**Results:**

HRV was reduced in MDD relative to controls, an effect associated with a medium effect size. MDD participants with comorbid generalized anxiety disorder displayed the greatest reductions in HRV relative to controls, an effect associated with a large effect size.

**Conclusions:**

Unmedicated, physically healthy MDD patients with and without comorbid anxiety had reduced HRV. Those with comorbid GAD showed the greatest reductions. Implications for cardiovascular risk reduction strategies in otherwise healthy patients with psychiatric illness are discussed.

## Introduction

Major depressive disorder (MDD) and cardiovascular disease (CVD) are leading burdens of disease worldwide, and there is increasing recognition that the two are related. Up to 50% of CVD patients suffer from depression [1], and depression increases the risk for cardiac mortality two to four times, irrespective of cardiac disease history [2]–[5]. Heart rate variability (HRV) – an index of the beat-to-beat changes in heart rate – is one candidate mechanism underlying the link between depression, CVD, and sudden cardiac death (SCD) [6], [7]. HRV is mediated by the parasympathetic and sympathetic nerves and reflects the capacity for the parasympathetic inhibition of autonomic arousal. Increased HRV reflects a healthy autonomic nervous system that is able to respond to changing environmental circumstances [8], [9]. By contrast, decreased HRV is a marker of autonomic inflexibility [10] and ill-health [11], that may precede more systemic problems such as inflammatory-mediated atherosclerosis and ventricular fibrillation [6], especially in younger samples as reported on here.

Research has shown that depressed patients with CVD display lower HRV than non-depressed patients [7]. There has been less examination of the impact on HRV of depression *without* comorbid physical illness. Using meta-analysis, we recently reported HRV reductions in depressed patients without CVD [12]. We interpreted these findings in the context of the polyvagal theory, which highlights a role for the autonomic nervous system in the somatomotor deficits and social impairment frequently observed in depression. [8] However, findings on the impact of MDD on HRV have been inconsistent; in particular, Licht et al. have reported that HRV reductions are driven by medication effects alone [13], [14]. However, reviews [15], [16] have argued that these inconsistencies point to the need to control for (1) physical illness such as CVD and diabetes; (2) medication status, which clearly impacts on HRV, but needs to be distinguished from the underlying effects of depression; (3) the presence of comorbid anxiety; and (4) to select participants to avoid the need to “control” for confounding variables using ANCOVA when participants are not randomly allocated to groups. These four issues underpin core methodological aspects of the present study, which are essential to isolate the effects of depression and comorbid anxiety on HRV.

Another body of evidence suggests that anxiety – a condition frequently comorbid with MDD in more than 60% of cases [17] – rather than depression contributes to the reductions in HRV [10], [15], [18] and CVD [19]. Intriguingly, patients with current anxiety disorders display an almost threefold increase in the prevalence of CVD, while no associations have been observed for depressive disorders without comorbidity [19]. Although low HRV has been reported in panic disorder (PD) and post-traumatic stress disorder (PTSD) [18], studies have seldom examined the impact of generalized anxiety disorder (GAD) on HRV [20]. While reduced HRV in PD [18] is consistent with the autonomic features characteristic of panic attacks, reduced HRV in GAD [10] may be driven by pre-attentive biases for threat information. It remains to be determined which comorbid anxiety disorders have the greatest impact on HRV, an important physiological marker of cardiovascular risk [6].

This study examined the impact of MDD and comorbid anxiety disorders on HRV to identify psychiatric indicators for cardiovascular risk reduction. Our hypothesis was that HRV would be reduced in MDD patients relative to age- and sex-matched controls in an independent, physically healthy, and unmedicated sample. Further, we sought to determine whether MDD without comorbidity, MDD with GAD or MDD with PD and/or PTSD display the greatest reductions in HRV. This is an important issue given the frequent comorbidity of MDD with anxiety disorders and competing accounts on the impact that anxiety with specific features (arousal in PD and PTSD versus apprehension or worry in GAD) may have on HRV [10], [18].

## Methods

### Participants

Seventy-three patients with a primary diagnosis of MDD and 94 age- and sex-matched controls were included in this study. Participants were recruited from the general community via self-referral from advertisements and collaborating clinicians. We obtained their data from the Brain Resource International Database (BRID [21]; www.brainresource.com). The study was approved by University of Sydney, Sydney West Area Health Service, University of Adelaide and Flinders University human research ethics committees, and all participants provided written informed consent in accordance with the Australian National Health and Medical Research Council guidelines.

Diagnoses were made by trained and supervised research officers using the Mini-International Neuropsychiatric Interview (MINI [22]) and the severity of clinical depression was assessed using the structured interview guide for the Hamilton Depression Rating Scale (SIGH-D [23]) (M = 20.29, SD = 4.34). Controls were recruited through community advertising and were excluded if they self-reported a history of psychiatric illness. Controls were further screened for an Axis 1 disorder using the Somatic and Psychological Health Report Questionnaire (SPHERE-12 [24]). The SPHERE-12 is a self-report screening tool for common mental disorders with acceptable validity and reliability [24], [25]. All participants completed the Depression Anxiety and Stress Scales (DASS-42 [26]), a reliable and valid self-report measure of depression, anxiety, and stress severity [26], [27] (Tables 1 and 2).

**Table 1:** MDD and Control Group Characteristics (mean, S.D.).

Variable	MDD Group (n = 73)	Control Group (n = 94)	*P* value
Age (SD)	36.5 (11.5)	35.7 (11.2)	0.64
Sex			
Male, %	41.1	44.7	0.64
Female, %	58.9	53.3	
DASS-42			
Depression (SD)	27.2 (9.4)	2.1 (2.4)	<0.001
Anxiety (SD)	13 (10.9)	1.2 (1.8)	<0.001
Stress (SD)	22.2 (9.8)	4.5 (4.5)	<0.001
Body Mass Index	25.3 (4.6)	25.8 (5.4)	0.56
AUDIT-C	2 (2.9)	2.4 (3)	0.49

Abbreviations: MDD = major depressive disorder; GAD: generalised anxiety disorder; PD: panic disorder; PTSD: posttraumatic stress disorder; DASS-42: depression, anxiety and stress scales; SIGH-D: Structured Interview Guide for the Hamilton Depression Rating Scale, AUDIT-C = alcohol use disorder identification test consumption subscale.

**Table 2:** Anxiety Subgroup Characteristics (mean, S.D.).

Variable	MDD with GAD (n = 24)	MDD with PD and/or PTSD (n = 14)	MDD without anxiety (n = 24)
Age (SD)	36.6 (11.7)	34.1 (12.1)	36.4 (10.6)
Sex			
Male, %	45.8	50	58.3
Female, %	54.2	50	41.7
DASS-42			
Depression (SD)	27.2 (11.5)	28.4 (7.9)	27.5 (8.9)
Anxiety (SD)	15.6 (10.6)	20 (11.5)	6.6 (6.1)
Stress (SD)	24.4 (10.2)	26.8 (7.2)	18.3 (9.8)
SIGH-D	21.5 (3.8)	20.3 (3)	18.5 (5.3)
Body Mass Index	24.2 (3.6)	27.5 (4.4)	25.8 (5.2)
AUDIT-C	1.5 (2.4)	2.2 (3.2)	2 (2.9)

Participants had no history of drug addiction, alcoholism, brain injury, loss of consciousness, stroke, neurological disorder, or other serious medical conditions (e.g., CVD and diabetes). All participants were free from anti-depressant medication for at least five half-lives (70% of whom were drug-naive). Twenty-four participants with MDD had comorbid GAD without other diagnosis (“anxious apprehension”). Fourteen participants with MDD had either comorbid PD (n = 7), PTSD (n = 4) or both (n = 3) (“anxious arousal”). No patient with comorbid PD and/or PTSD had GAD.

### Procedure

Participants were seated in a sound- and light-controlled room at 24°C and two 2-minute electrocardiogram (ECG) recordings were collected during resting state. The data was sampled at 500 Hz, with 22-bit resolution digitization using a Compumedics Neuroscan NuAmps amplifier and SCAN software, version 4.3. Experimental studies have shown that 2-minute recordings are predictive of coronary heart disease and mortality [28], [29] and can provide a more accurate picture of physiological changes than longer-term recordings (which inevitably introduce variations from a physiologically fixed state under laboratory conditions) [30], [31].

### Data Analysis

All data was manually inspected prior to analysis: data quality was high, consistent with resting-state recording conditions. ECG was analyzed using custom-developed software to perform semi-automated pre-processing to remove noise from the ECG, allowing for the identification of the R-peaks based on established methods [32]. Cleaned R–R interval data was then imported into Kubios software (available at: http://kubios.uku.fi) for calculation of time, frequency and nonlinear measures. The two time-domain measures calculated on the R–R intervals include the standard deviation of R–R intervals (SDNN), and the square root of the mean squared differences between successive R–R intervals (RMSSD), a measure based on comparisons of lengths of adjacent cycles. Two frequency domain measures were quantified: high frequency (HF; 0.18–0.40 Hz), a measure of parasympathetic activity; and the LF/HF ratio, a measure of sympathovagal balance. We also examined two nonlinear domain measures: the standard deviation of the Poincaré plot perpendicular to the line of identity (PCSD1), and detrended fluctuation analysis (short-fluctuation slope, DFAα1). The Poincaré graph plots each R–R interval as a function of the next R–R interval, and the PCSD1 measure represents the short-term variability of the nonlinear dynamic system and reflects parasympathetic nervous system activity. DFA measures the correlation within the signal, and the DFAα1 measures the short-range correlation between successive R–R intervals. White Gaussian noise (a totally random signal) is reflected in a value of 0.5; a Brownian noise signal (a signal in which higher frequencies display decreased power) is reflected in a value of 1.5 [33].

Statistical analysis was conducted using predictive analytics software (PASW) statistics, version 18. Independent-samples t-tests on controls versus MDD, and one-way ANOVAs on controls versus the three MDD groupings were conducted to determine differences on age, gender, body mass index (BMI), and alcohol use (Alcohol Use Disorders Test – Consumption Scale; AUDIT-C [34]). ANOVAs were followed up by Tukey's post-hoc tests. Statistics were conducted on SIGH-D [23] and DASS-42 [26] to confirm the validity of diagnostic groupings and determine whether there were differences between patient groupings in disorder severity (SIGH-D and DASS-42 depression scores). A multivariate analysis of variance (MANOVA) was performed to determine whether there were differences between MDD patients and controls on an interpretable composite of HRV variables across time, frequency, and nonlinear domains, and to provide a control for multiple comparisons. A significant Pillai's trace effect for Group was followed by univariate analyses of variance (ANOVAs) to identify which measures contribute to a significant multivariate effect. Effect size measures (Cohen's *d*) relating to the difference between patient groups and controls were determined.

## Results

### Group Characteristics

**Figure 1:**
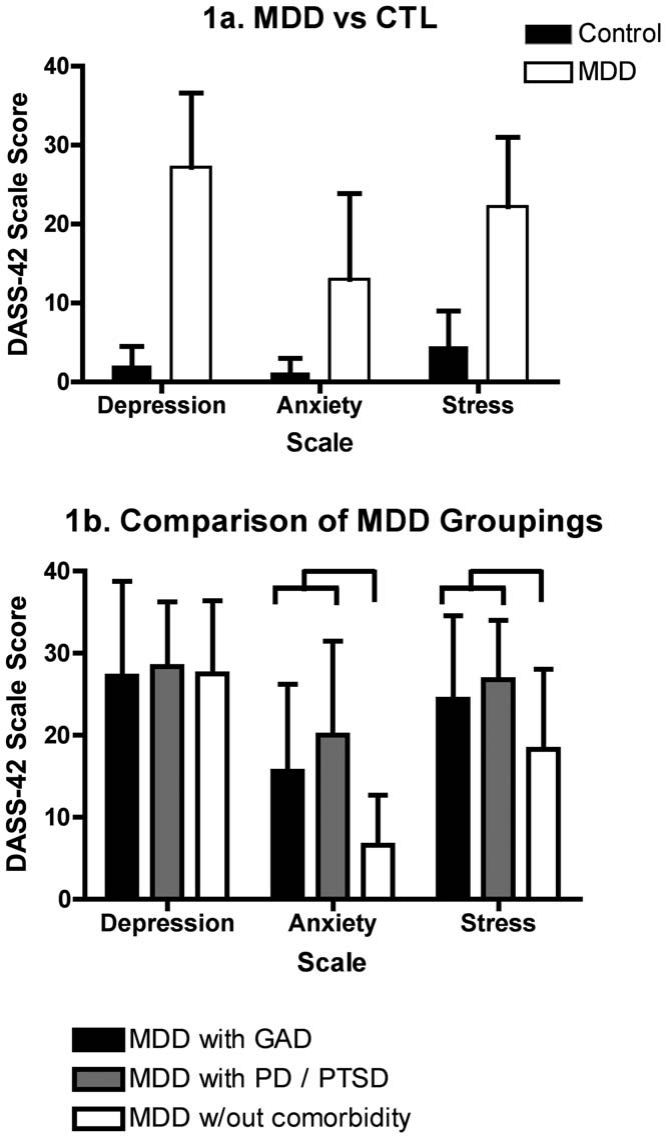
DASS-42 scale scores (mean ± standard deviation) for MDD vs CTL (1a) and MDD groupings.

Table 1, Table 2 and Figure 1 present participant characteristics. There were no group differences on age, gender, BMI, or alcohol use. As expected, all patient groupings differed from controls on depression (p<.001), anxiety (p<.001) and stress (p<.001) scales (Table 1, Figure 1a). No differences were observed between different patient sub-groupings on SIGH-D or self-reported patient DASS-42 depression severity (Table 2, Figure 1b), nor between MDD patients with GAD and those with comorbid PD and/or PTSD on self-reported measures of anxiety or stress. Patient groups with comorbid anxiety differed from those without anxiety on DASS-42 measures of anxiety (p<.001) and stress (p<.03), but not depression (Table 2, Figure 1b).

### Heart Rate Variability

**Figure 2:**
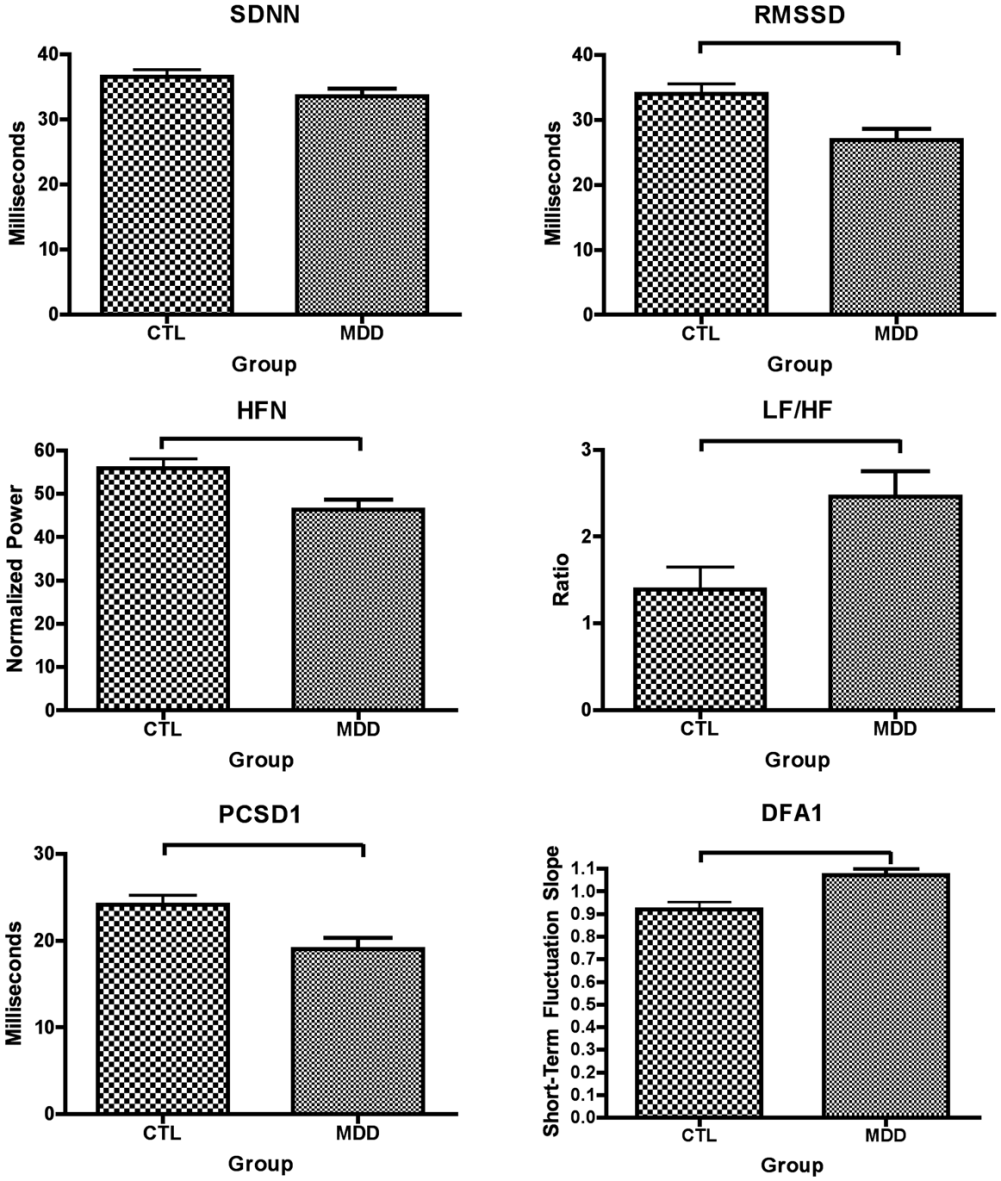
Time (row 1), frequency (row 2) and non-linear (row 3) domain measures of HRV in unmedicated patients with major depressive disorder (MDD) relative to controls (CTL).

**Figure 3:**
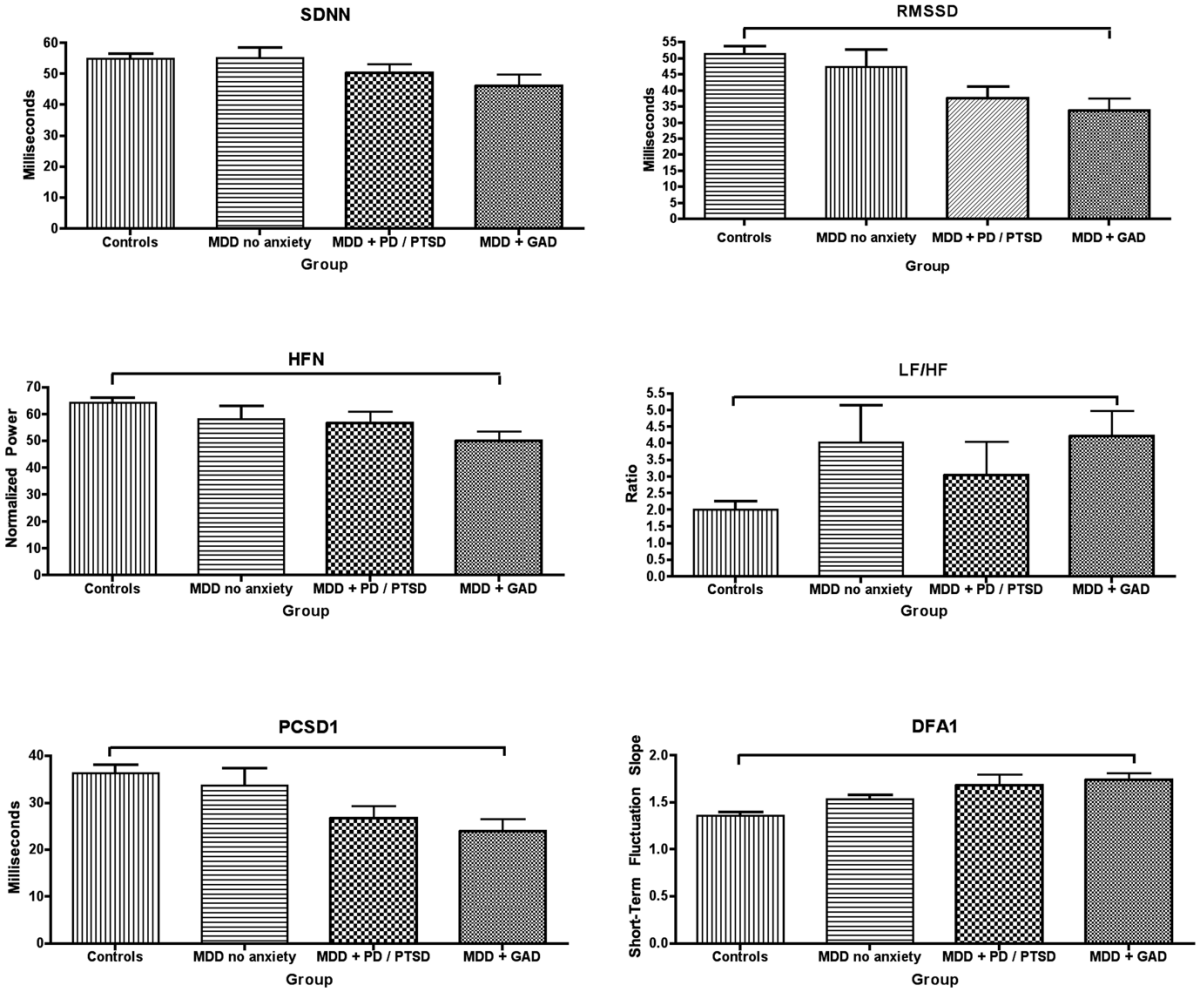
Time (row 1), frequency (row 2) and non-linear (row 3) domain measures of HRV in controls, MDD without anxiety, MDD with comorbid PD and/or PTSD and MDD patients with GAD.

Our hypothesis that HRV is reduced in MDD relative to controls (CTL) was confirmed by an overall MANOVA, including all HRV measures [F(12,154) = 2.992, p = .001, partial eta squared = .189] (Figure 2). Strikingly, the partial eta squared value indicated that 19% of the variance in HRV is attributable to MDD. MANOVA also identified significant differences between MDD groups with comorbid GAD (anxious *anticipation*), comorbid PD and/or PTSD (anxious *arousal*), MDD without anxiety comorbidity, and healthy control groups [F(36,417) = 1.780, *p* = .005, partial eta squared = .131]. Figure 3 shows linear decreases in all measures of HRV (with the exception of the LF/HF ratio); these decreases are most pronounced in MDD patients with comorbid GAD, followed by patients with comorbid PD and/or PTSD, and MDD patients without anxiety relative to controls. ANOVAs are reported below to identify which measures contribute to these significant multivariate effects.

### Heart Rate Variability: MDD Versus Control Groups

These results are shown in Figure 2. In time-domain measures, the MDD group displayed reductions in RMSSD (F (165) = 9.41, p = 0.003, Cohen's *d* = −0.48) and SDNN (at trend levels) (F (165) = 3.57, p = 0.06, Cohen's *d* = −0.29). In frequency-domain measures, the groups differed on HF (F (165) = 8.712, p = 0.004, Cohen's *d* = −0.46) and LF/HF ratio (F (165) = 7.71, p = 0.006, Cohen's *d* = 0.42). Although the LF/HF ratio was increased in MDD relative to CTL, this finding is interpreted as an increase in sympathetic relative to parasympathetic activity in MDD, consistent with a decrease in HRV. In nonlinear-domain measures, the groups differed on PCSD1 (F (165) = 9.35, p = 0.003, Cohen's *d* = −0.48) and DFAα1 (F (165) = 11.30, p = 0.001, Cohen's *d* = 0.52). Although the DFAα1 measure is increased in MDD relative to CTL, this finding indicates that the MDD group displays a less random signal than CTL, consistent with a reduction in HRV.

### Heart Rate Variability: All Four Groups

These results are shown in Figure 3. In time-domain measures, ANOVA revealed a statistically significant difference for RMSSD [F(3, 152) = 4.8, *p* = .003, η^2^ = .09] and SDNN (at trend levels) [F(3, 152) = 2.58, *p* = .06, η^2^ = .05]. Post-hoc comparisons revealed that RMSSD is reduced in MDD patients with GAD (p = .004, Cohen's *d* = .835). No other patient groups differed from each other or from controls.

In frequency-domain measures, ANOVA revealed a statistically significant difference for HF [F(3,152) = 4.6, *p* = .004, η^2^ = .08] and LF/HF ratio [F(3, 152) = 4.1, *p* = .008, η^2^ = .08]. Post-hoc comparisons revealed that HF (p = .003, Cohen's *d* = .854) was reduced and LF/HF ratio (p = .028, Cohen's *d* = .943), increased in MDD patients with GAD, relative to controls. MDD patients with no anxiety also displayed increased LF/HF ratio relative to controls at trend levels (p = .051, Cohen's *d* = .661). No other significant differences were observed.

In nonlinear-domain measures, ANOVA revealed a statistically significant difference for PCSD1 [F(3, 152) = 4.7, *p* = .003, η^2^ = .09] and DFAα1 [F(3, 152) = 6, *p* = .001, η^2^ = .11]. Post-hoc comparisons revealed that PCSD1 (p = .004, Cohen's *d* = −.834) decreased and DFAα1 ratio (p = .002, Cohen's *d* = .958) increased in MDD patients with GAD, relative to controls. MDD patients with PD and/or PTSD also displayed a statistical trend towards increased DFAα1 ratio (p = .063, Cohen's *d* = .744) No other significant differences were observed.

## Discussion

Recent debate [35]–[38] has focused on inconsistent findings concerning whether HRV is reduced in depression *per se* [12], or whether these reductions are driven by antidepressant medication [14]. We found that HRV is reduced in unmedicated patients diagnosed with MDD without CVD across a variety of measures, an effect associated with a medium effect size. Other lines of evidence [10], [15] have suggested that anxiety rather than depression drives the reported HRV reductions in MDD, although until now, specific comorbid anxiety disorders that have the greatest impact on HRV have not been identified. Our findings indicate that HRV is most reduced in comorbid GAD, and that these reductions were not due to depression severity – all MDD groupings rated similarly on levels of depression severity – or other potential confounding variables such as age, BMI, alcohol use, and physical illness including diabetes and CVD. The advantages of the current study are (i) the exclusion of medicated patients and those with comorbid physical illness, (ii) the subgrouping of patients with and without anxiety disorders, (iii) the careful selection of participants to avoid the need for ANCOVA and (iv) the inspection of a variety of HRV variables.

GAD is characterized by anxious apprehension and worry, involving pre-attentive biases to threat information, and rigid and inflexible response patterns [39]. The findings of the current study indicate that depressed patients with a secondary diagnosis of GAD had the largest decreases in HRV, and the large effect sizes observed highlight the clinical relevance of this effect. A recent study on HRV [13], examined the impact of current anxiety disorders (n = 1159), including GAD (38.2% of the sample), PD (55.4%) and social phobia (55.5%), relative to controls (n = 616), reporting that while patients had lower SDNN and respiratory sinus arrhythmia, no differences were observed between the anxiety disorders, and the effects disappeared after adjustment for antidepressant use. We have raised a number of concerns over the findings reported by Licht and colleagues elsewhere [16], [35], [36], and simply note here that while the sample size of the study is impressive, a number of methodological issues may account for their findings. The current study provides an important extension to prior work [10], [20], indicating that comorbid anxiety is associated with the greatest reductions in HRV and that patients with comorbid GAD may benefit most from cardiovascular risk reduction strategies such as behavioural modification, exercise and addressing other risk factors (e.g. smoking and moderating alcohol use). While it is possible that patients characterized by anxious arousal may display greater reductions during emotional challenge (e.g., stress), short-term resting-state measures of HRV better reflect intrinsic HRV [30].

**Figure 4:**
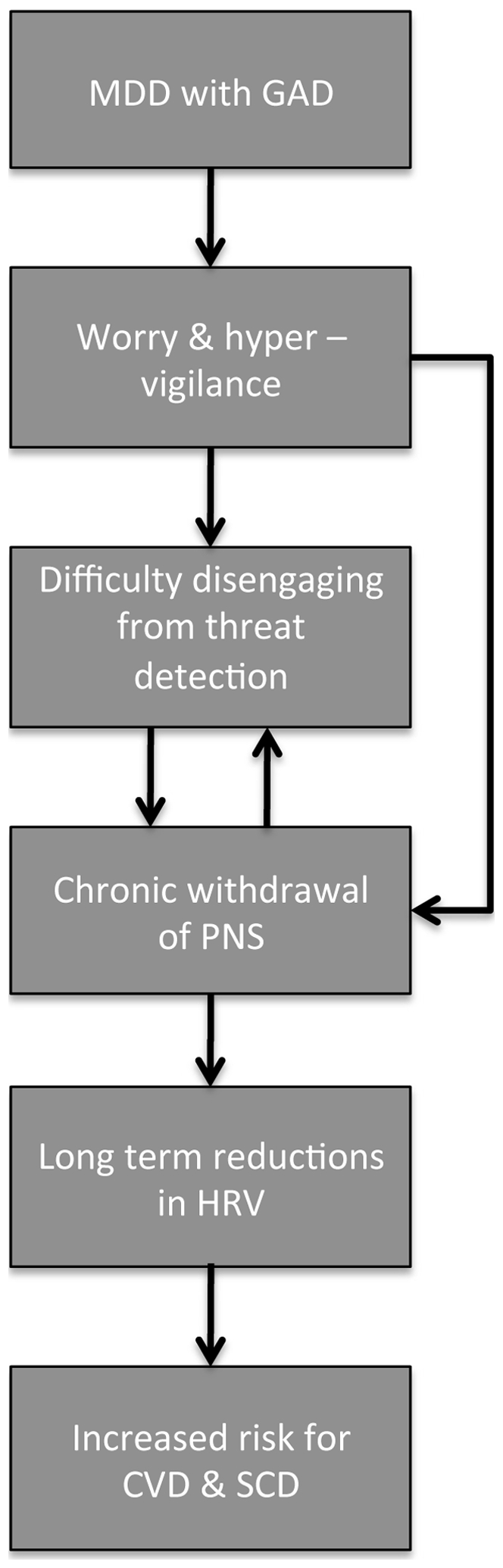
Proposed model for reduced heart rate variability (HRV) in patients with major depressive disorder (MDD) and comorbid generalised anxiety disorder (GAD). These patients typically experience worry and hypervigilance to threat, leading to a chronic withdrawal of the parasympathetic nervous system (PNS) and long-term reductions in HRV, placing these patients at an increased risk for cardiovascular disease (CVD) and sudden cardiac death (SCD).

There are a number of explanations for why HRV may be reduced in depression and in those with comorbid GAD in particular. One potential explanation proposed for the link between anxiety, HRV and CVD is the inability to disengage threat detection, which serves to perpetuate worry and hypervigilance, even when no real threat exists [10]. Reductions in parasympathetic tone may be a consequence of reduced activation within the central autonomic network (CAN) [9], a network of brain regions that control a variety of visceromotor, neuroendocrine, and behavioral responses critical for goal-directed behavior and behavioral flexibility. Neuroimaging studies report that decreases in HRV are associated with concomitant decreases in activation in a number of brain regions, including the right superior prefrontal (BA 8, 9), left rostral anterior cingulate (BA 24, 32), right dorsolateral prefrontal (BA 46), and right parietal (BA 40) cortices [9]. The neurovisceral integration model highlights a role for the prefrontal cortex – particularly the orbitofrontal and medial prefrontal cortices – in vagally mediated cardiovascular control. HRV appears to index the functional integrity of the CAN and may underpin successful adjustment, self-regulation, and psychological flexibility in response to environmental demands [9]. Therefore, the reductions observed in MDD patients with comorbid GAD may be associated with prolonged prefrontal inactivity, disinhibition of the central nucleus of the amygdala, and activation of medullary cardioacceleratory circuits [9]. Our results suggest that patients with MDD and comorbid GAD, a disorder characterized by worry and hypervigilance, may have difficulty disengaging from threat detection, which may lead to a chronic withdrawal of parasympathetic nervous system activity, overactivation of sympathetic nervous system activity, and long-term reductions in HRV, subsequently increasing the risk for CVD and SCD (Figure 4).

It is possible that the observed HRV reductions were due to potential lifestyle factors not assessed in the current study, such as smoking or low physical activity levels in the depressed patients. While it is possible that these factors could explain the differences observed between MDD patients and controls, it is unlikely they could explain the differential effects observed for patient sub-groupings relative to controls. Recent work also highlights a relationship between HRV and inflammatory markers [40], [41] such as interleukin-6 (IL-6) and C-reactive protein (CRP). This is important because inflammation also plays a role in the pathogenesis of CVD. While we did not collect this information, our study reports on a much younger cohort than these studies, and as age is a well-established cardiovascular disease risk factor associated with an increasing inflammatory burden [42], it is less likely that changes in these parameters would be observed in our sample. Regardless, autonomic imbalance is considered to be a final common pathway to increased morbidity and mortality [43]. While acetylcholine released from the vagus inhibits proinflammatory cytokine production, reduced vagal tone (reflected as reductions in HRV) leads to a heightened inflammatory state, and development of CVD [6], [44]. We suggest therefore that changes in HRV may precede the development of these other critical, ‘downstream’ risk markers, particularly in younger samples.

In conclusion, this study has provided strong evidence that clinicians should consider comprehensive cardiovascular risk-reduction strategies for MDD patients with comorbid GAD in particular. Further research examining intermediate (HRV) (as well as final endpoints such as mortality rate) is required to confirm whether such strategies are needed for patients with MDD alone, and MDD with comorbid PD and/or PTSD.
